# Decreased enrollment in breast cancer trials by histologic subtype: does invasive lobular carcinoma resist RECIST?

**DOI:** 10.1038/s41523-021-00348-z

**Published:** 2021-10-25

**Authors:** Mary Kathryn Abel, Michelle E. Melisko, Hope S. Rugo, A. Jo Chien, Italia Diaz, Julia K. Levine, Ann Griffin, Joseph McGuire, Laura J. Esserman, Hala T. Borno, Rita A. Mukhtar

**Affiliations:** 1 University of California, San Francisco School of Medicine, San Francisco, CA USA; 2 University of California, San Francisco, Department of Surgery, San Francisco, CA USA; 3 University of California at San Francisco, Division of Hematology/Oncology, San Francisco, CA USA; 4 University of California, Davis School of Medicine, Sacramento, CA USA; 5 Lobular Breast Cancer Alliance, Venice, CA USA; 6 Helen Diller Family Comprehensive Cancer Center, San Francisco, CA USA

**Keywords:** Breast cancer, Breast cancer

## Abstract

Enrollment in metastatic breast cancer trials usually requires measurable lesions, but patients with invasive lobular carcinoma (ILC) tend to form diffuse disease. We found that the proportion of patients with metastatic ILC enrolled in clinical trials at our institution was significantly lower than that of patients with invasive ductal carcinoma (IDC). Possible links between requiring measurable disease and decreased enrollment of ILC patients require further study to ensure equitable trial access.

The need for unified criteria and a common language to evaluate treatment efficacy in oncologic clinical trials led to recommendations for standardized definitions from the World Health Organization^1^. These recommendations subsequently formed the basis for the Response Evaluation Criteria in Solid Tumors (RECIST), now one of the most common means of objectively measuring tumor response in therapeutic clinical trials^2,3^. Despite its widespread use, some have criticized RECIST because change in tumor size, the main response endpoint utilized, may not translate to overall survival difference^4^. Others have identified particular tumor types for which RECIST may not optimally capture treatment response^5^. ILC of the breast may be one such tumor type. ILC is the second most common type of breast cancer after IDC, accounting for 10–15% of all cases^6,7^. In the metastatic setting, ILC forms diffuse lesions in the gastrointestinal tract, peritoneal lining, leptomeninges, or pleura, with its characteristic lack of adhesion protein E-cadherin likely contributing to its diffuse growth pattern^6^.

Other tumor types for which investigators have identified challenges with RECIST utilize alternative disease-specific factors in clinical trial eligibility. For example, metastatic prostate cancer frequently presents with unmeasurable bone metastases. The Prostate Cancer Clinical Trials Working Group recommends utilization of disease-specific caveats to RECIST and investigation into surrogate biomarkers^8,9^. Similarly, modified RECIST criteria have been proposed for malignant mesothelioma, which has an ill-defined, diffuse growth pattern^10,11^. Currently, there are no special provisions for ILC in clinical trial enrollment criteria. In this report, we characterize the utilization patterns of RECIST or comparable measurable disease criteria among therapeutic, metastatic breast cancer trials registered in *clinicaltrials.gov*. We then employ a retrospective analysis to assess clinical trial enrollment patterns by breast histology and stage using the University of California, San Francisco (UCSF) Cancer Registry and an institutional clinical trials registry (OnCore clinical trials management system [CTMS]).

There were 160 actively recruiting, interventional clinical trials for stage IV breast cancer in *clinicaltrials.gov*. Fourteen behavioral and dietary supplement studies were excluded. Of the 146 remaining studies, 119 (81.5%) were drug trials. Overall, 104 (71.2%) required measurable disease for study participation. Of these 104 studies, 29 (27.9%) utilized RECIST in inclusion criteria; 22 (21.5%) utilized RECIST as an outcome measure, and 48 (46.2%) utilized RECIST in both inclusion criteria and outcome measures. Five (4.8%) studies used measurable or alternative size criteria. No trials explicitly restricted the study population by histology.

The UCSF Cancer Registry included 8679 patients, including 7320 (84.3%) IDC patients and 1359 (15.6%) ILC or mixed ILC/IDC patients. Most patients had stage I–III disease (*n* = 8187, 94.3%), whereas 492 (5.7%) had metastatic disease. There were 1511 individuals enrolled in OnCore CTMS, including 1304 (86.3%) patients with IDC and 207 (13.7%) patients with ILC or mixed ILC/IDC. Of these 1337 (88.5%) had stage I–III disease, and 174 (11.5%) had metastatic disease. In patients with early-stage disease, where RECIST is not typically used, there was no difference in the proportion of ILC patients enrolled in clinical trials versus in the cancer registry (Fig. 1). However, among those with stage IV disease, there was a significantly lower proportion of ILC patients in the OnCore CTMS than the cancer registry (9.2% versus 17.9%, *p* = 0.005). In contrast, patients with metastatic IDC were overrepresented in clinical trials compared with the cancer registry.

**Fig. 1:**
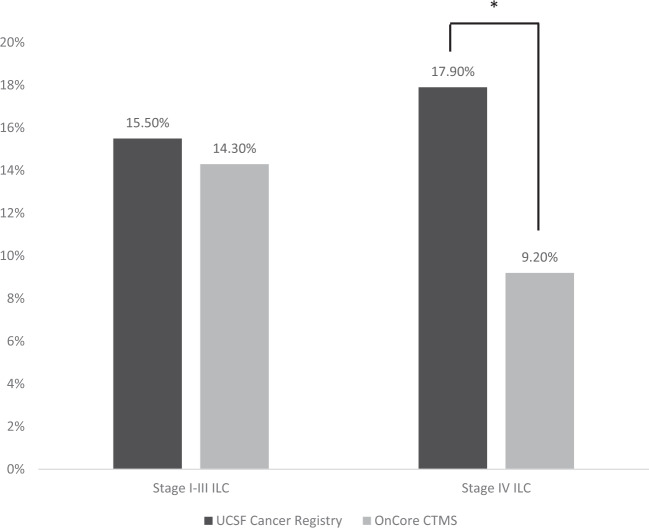
Proportion of patients with invasive lobular carcinoma in the UCSF Cancer Registry versus the OnCore Clinical Trials Management System (CTMS), stratified by stage. Patients with stage IV invasive lobular carcinoma (ILC) are significantly under-represented in the OnCore CTMS registry compared with the UCSF Cancer Registry. *Two-sample test of proportion: *p* = 0.005.

Overall, we found that the majority of stage IV breast cancer trials utilize RECIST and that patients with metastatic ILC are significantly less likely to be enrolled in clinical trials in an institutional database. These findings are important because clinical trial participation is associated with improved overall survival^12,13^. Although we cannot conclude from this work that high use of RECIST contributes to lower clinical trial enrollment rates for ILC patients, our findings warrant attention to this potential issue and further examination of larger datasets. We do hypothesize that ILC may represent another tumor type for which modified RECIST criteria may be useful. Apart from lacking E-cadherin, investigators have reported differences in tumor stroma between ILC and IDC tumors, with fewer CD34-positive fibroblasts, lower levels of tumor-infiltrating lymphocytes, and reduced desmoplastic reaction in ILC. Such differences may contribute to the lower sensitivity of imaging studies for ILC^14–16^. Alternative endpoints for treatment response in ILC could rely upon novel imaging tools targeting the high levels of estrogen receptor positivity, such as [18]F-fluoroestradiol PET imaging^17^. Patients with metastatic ILC have also been shown to have higher absolute levels of circulating tumor cells than those with metastatic IDC, suggesting that liquid biopsy might be a good indicator of disease response^18^.

Our analysis of CTMS and the cancer registry are restricted to a single institution, limiting its generalizability. Owing to its retrospective nature, our study lacked patient-level data regarding why individuals were not enrolled in trials, such as tumor receptor subtype. Although we cannot conclude from these analyses that lack of measurable disease resulted in decreased enrollment, these findings certainly should compel further investigation to ensure equal clinical trial opportunities for patients with ILC. As with other cancer types that form diffuse disease, modifications to RECIST or alternative endpoints specifically for ILC may be warranted.

## Methods

### Data sets

The *clinicaltrials.gov* registry, a web-based registry maintained by the National Library of Medicine and National Institutes of Health, was queried to identify trials from inception to July 2019. We used the search function to select the following factors: “stage IV breast cancer”, “actively recruiting”, and “interventional studies (clinical trials)”. Utilization of measurable disease criteria was defined as either (1) the explicit use of RECIST, (2) the definition of measurable disease by RECIST (imaging or physical exam that shows at least one measurable lesion with a minimum size in at least one diameter of ≥10 mm for lesions and ≥15 mm for lymph nodes), or (3) the explicit requirement for measurable disease without further qualification.

The University of California, San Francisco (UCSF) Cancer Registry of the Helen Diller Family Comprehensive Cancer Center was used to capture individuals with invasive breast cancer who received care at UCSF between 1 January 2000 to 31 December 2018. In addition, we utilized the OnCore CTMS to identify patients with invasive breast cancer who were enrolled in interventional, therapeutic clinical trials at UCSF during the same timeframe. Only patients with documented IDC, ILC, or mixed ILC/IDC histology were included. To assess differences between registry and clinical trial enrollment, we used two-sample tests of proportions stratified by histology (IDC versus ILC) and stage (I–III versus IV). Data were analyzed in Stata 14.2 (StataCorp LLC, College Station, TX, USA). The study was approved by the UCSF Institutional Review Board.

### Reporting summary

Further information on research design is available in the Nature Research Reporting Summary linked to this article.

## Supplementary information

Reporting Summary

## Data Availability

Aggregate data are available upon reasonable request with appropriate institutional review board approval.

## Code availability

Analysis codes are available from the corresponding author upon reasonable request.
